# Bicarbonate in diabetic ketoacidosis - a systematic review

**DOI:** 10.1186/2110-5820-1-23

**Published:** 2011-07-06

**Authors:** Horng Ruey Chua, Antoine Schneider, Rinaldo Bellomo

**Affiliations:** 1 Department of Intensive Care, Austin Health, Melbourne, Victoria, Australia; 2 Australia and New Zealand Intensive Care (ANZIC) - Research Centre, Monash University, Melbourne, Victoria, Australia

## Abstract

**Objective:**

This study was designed to examine the efficacy and risk of bicarbonate administration in the emergent treatment of severe acidemia in diabetic ketoacidosis (DKA).

**Methods:**

PUBMED database was used to identify potentially relevant articles in the pediatric and adult DKA populations. DKA intervention studies on bicarbonate administration versus no bicarbonate in the emergent therapy, acid-base studies, studies on risk association with cerebral edema, and related case reports, were selected for review. Two reviewers independently conducted data extraction and assessed the citation relevance for inclusion.

**Results:**

From 508 potentially relevant articles, 44 were included in the systematic review, including three adult randomized controlled trials (RCT) on bicarbonate administration versus no bicarbonate in DKA. We observed a marked heterogeneity in pH threshold, concentration, amount, and timing for bicarbonate administration in various studies. Two RCTs demonstrated transient improvement in metabolic acidosis with bicarbonate treatment within the initial 2 hours. There was no evidence of improved glycemic control or clinical efficacy. There was retrospective evidence of increased risk for cerebral edema and prolonged hospitalization in children who received bicarbonate, and weak evidence of transient paradoxical worsening of ketosis, and increased need for potassium supplementation. No studies involved patients with an initial pH < 6.85.

**Conclusions:**

The evidence to date does not justify the administration of bicarbonate for the emergent treatment of DKA, especially in the pediatric population, in view of possible clinical harm and lack of sustained benefits.

## Introduction

Diabetic ketoacidosis (DKA) is a serious medical emergency resulting from relative or absolute insulin deficiency and the unopposed action of counter-regulatory hormones, such as glucagon, cortisol, and catecholamines [1]. The hepatic metabolism of free fatty acids generates ketoanions, such as beta-hydroxybutyrate and acetoacetate [2,3]. Impaired tissue perfusion due to volume contraction and the adrenergic response to the often severe underlying precipitating illness result in lactate production [4]. Acute kidney injury leads to accumulation of other unmeasured anions, such as sulphate, urate, and phosphate [5]. All these, together with hyperchloremia which predominates during the recovery phase of DKA [6], contribute to the development of acidemia, which often is severe [7,8].

Experimental studies suggest that metabolic acidemia can impair myocardial contractility, reduce cardiac output, affect oxyhemoglobin dissociation and tissue oxygen delivery, inhibit intracellular enzymes, such as phosphofructokinase, alter cellular metabolism, and result in vital organ dysfunction [9-12]. Thus, the target of therapy in DKA has historically placed importance on the rapid reversal of acidemia, in addition to the correction of dehydration and insulin deficiency.

As a result of the physiological paradigm, correction of severe acute acidemia with intravenous bicarbonate to attenuate the deleterious effects continues to be utilized by some practitioners. This approach has received wide acceptance in the past, but based on currently available evidence, and concerns about the potential adverse effects in children and adults, the administration of bicarbonate in DKA requires re-examination.

The objective of this systemic review was to examine the medical evidence to date, on the administration of bicarbonate versus no bicarbonate, in the emergent treatment of severe acidemia in pediatric and adult patients with DKA, with regards to the physiological and clinical efficacies and harms of this intervention.

## Methods

### Information source

Literature search was performed using the PUBMED database. The list of potentially relevant article titles and abstracts was generated by using the keywords, "bicarbonate" AND "diabetic ketoacidosis."

### Study selection and eligibility criteria

Two investigators (HC and AS) independently reviewed the article titles and abstracts. The following exclusion criteria were first applied: 1) review articles; 2) commentaries, letters, or editorials; 3) non-English articles; 4) animal studies; 5) all articles not related to acid-base issues, bicarbonate use, or cerebral edema in DKA; 6) publications before 1960.

The remaining papers were deemed relevant if they fulfilled the following inclusion criteria:

1. Population: Both adult and pediatric populations with diagnosis of DKA

2. Intervention: Intravenous sodium bicarbonate therapy

3. Comparator: Bicarbonate administration versus no bicarbonate for the emergent treatment of diabetic ketoacidosis

4. Outcome: Primary outcomes are the difference in mortality and duration of hospitalization. Secondary outcome is a combination of various physiological and clinical outcomes. Physiological outcomes include resolution of acidosis and ketosis, insulin sensitivity and glycemic control, potassium balance, tissue oxygenation, and cerebrospinal fluid (CSF) acidosis. Clinical outcomes include hemodynamic stability and neurological outcomes, including that of cerebral edema (CE)

5. Study type: All trials, including randomized and nonrandomized case-control studies, as well as case reports and series were selected.

Two investigators (HC and AS) reviewed all remaining papers in entirety after the application of the above-mentioned criteria. A third independent investigator (RB) adjudicated any disagreements regarding paper inclusion.

## Results

### Search results

**Figure 1:**
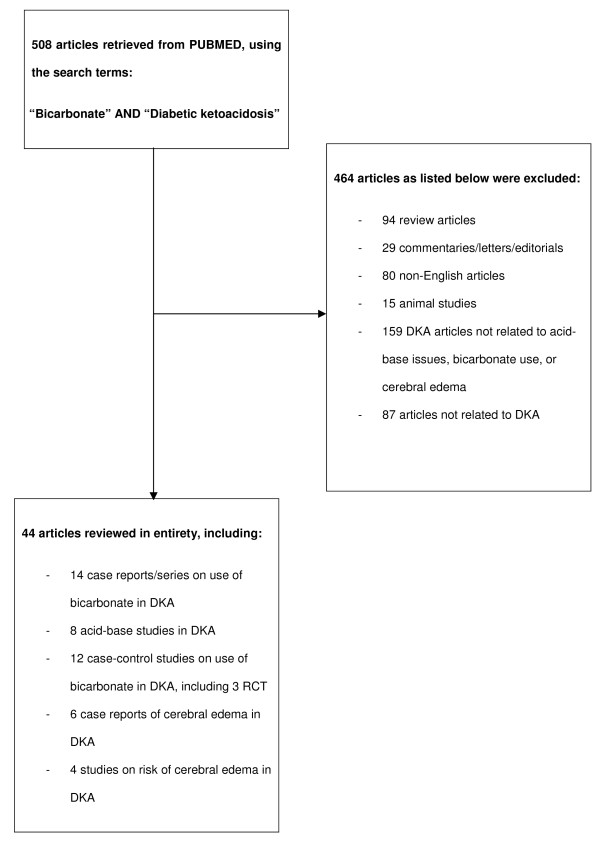
**Overview of study selection process**.

The systematic search identified 508 potentially relevant citations. Following application of the inclusion and exclusion criteria, 44 articles were eventually selected and the full manuscripts were reviewed. The selection process is illustrated in Figure 1.

### Study characteristics

Twelve publications were case-controlled studies on bicarbonate administration versus no bicarbonate in DKA. Of these, two studies were nonblinded randomized controlled trials (RCT) [13,14], and one study was a double-blind RCT [15]. A total of 73 adult patients were included in these three RCTs. The remaining nine studies were nonrandomized, prospective, or retrospective studies, which include six adult studies [16-21], two involving both adult and pediatric patients [22,23], and one pediatric study [24]. No RCTs have been performed in the pediatric cohort, and no trials have examined bicarbonate treatment in DKA patients with an admission pH < 6.85. In addition, four pediatric nonrandomized prospective and retrospective studies investigated the association between bicarbonate administration in DKA and risk of CE [25-28]. There were no similar studies in the adult DKA cohort.

### Study threshold for and dose of bicarbonate

In Table 1 we summarized the threshold for bicarbonate administration in various studies, which includes the initial degree of acidemia and base deficit [4,13-24,29-36]. There is heterogeneity of initial pH threshold for bicarbonate therapy, which has become more stringent over the years, from pH < 7.20 in the past to pH < 7.00.

**Table 1:** Degree of baseline acidemia and base deficit in DKA patients with bicarbonate administered

Reference	Population	Nature of study	Mean initial blood indices		
			pH	Base deficit	Bicarb (mmol/L)
Addis 1964 [29]	A. (N = 3)	Case series	6.94	Mostly unavailable	
Kuzemko 1969 [30]	P. (N = 6)	Case series	7.05	23	8.0
Zimmet 1970 [4]	A. (N = 11)	Case series	7.09	24	4.4
Soler 1972 [22]	A+P. (N = 18)	Prospective C-C	< 7.2	NR	< 10.0
Krumlik 1973 [31]	P. (N = 27)	Case series	7.05	NR	7.6
Soler 1974 [32]	A. (N = 1)	Case report	6.85	NR	6.0
Munk 1974 [16]	P. (N = 5)	Prospective C-C	7.05	22	8.7
Assal 1974 [23]	A+P. (N = 9)	Retrospective C-C	7.06	NR	5.6
Keller 1975 [33]	A. (N = 58)*	Case series	< 7.2	NR	NR
Reddy 1977 [34]	P. (N = 19)	Case series	7.07	NR	6.5
Lutterman 1979 [17]	A. (N = 12)	Retrospective C-C	6.89	NR	NR
Lever 1983 [18]	A. (N = 52)	Retrospective C-C	6.94-7.00^†^	NR	3.4-4.3^†^
Hale 1984 [13]	A. (N = 16)	RCT	6.85	NR	7.0
Morris 1986 [14]^‡^	A. (N = 10)	RCT	7.03	NR	3.6
Gamba 1991 [15]^‡^	A. (N = 9)	RCT (DB)	7.05	NR	2.9
Okuda 1996 [19]	A. (N = 3)	Prospective C-C	6.98	NR	2.0
Green 1998 [24]	P. (N = 57)	Retrospective C-C	7.02	40	NR
Viallon 1999 [20]	A. (N = 24)	Retrospective C-C	6.93	NR	3.1
Latif 2002 [21]	A. (N = 4)	Retrospective C-C	6.85	NR	NR
Kamarzaman 2009 [35]	A. (N = 1)	Case report	6.27	41	4.0
Guneysel 2009 [36]	A. (N = 1)	Case report	6.82	27	8.4

A = adults; P = pediatrics; N = number of patients who received bicarbonate, if breakdown available; C-C = case-control; RCT = randomized, controlled trial; DB = double-blinded; Bicarb = bicarbonate level; NR = not reported

*Breakdown of patients with or without bicarbonate administered not provided

^†^Mean values provided separately for two different study centers

^‡^Patients with initial pH < 6.9 were excluded from the RCT

Dosing methods vary widely with study design and physician preference, and these are summarized in Table 2. Concentrated bicarbonate dosing based on calculations using predictive formulas incorporating base deficit [37,38] results in a tendency for over-correction and alkalosis [29,30]. Aiming for a more modest and intermediate pH target with bicarbonate dose less than half of that predicted, or dose titrated based on pH severity, were some of the variable approaches adopted subsequently by investigators [4,23]. Consequentially, the average bicarbonate dose reported in studies appears to have decreased over the years to an overall amount of 120-150 mmol for adults and 2 mmol/kg for children.

**Table 2:** Summary of bicarbonate dose administered in case series and studies

Reference	Nature of Study	Dose of bicarbonate given (mean)			Dose Estimation	Timing (range)
		Conc (%)	Total (mM)	Wt-adj (mM/kg)		
Addis 1964 [29]	CS	8.4	413	NR	based on calculated dose	150 initial, and rest
						over 1.5 to 12 hr
Kuzemko 1969 [30]^P^	CS	8.4	255	NR	based on calculated dose	over 3 to 32 hr
Zimmet 1970 [4]	CS	NR	185	NR	based on pH severity	within initial 4 hr
					(≈ half of calculated dose)	
Soler 1972 [22]^AP^	PrC	1.0	200 - 400^†^	NR	NR	NR
Krumlik 1973 [31]^P^	CS	7.5	115 (3.3/kg) to reach pH ≥ 7.2		based on calculated dose	half over 30 min,
			144 (3.9/kg) to reach pH ≥ 7.3			rest over 2 hrs
Munk 1974 [16]^P^	PrC	NR	130	2.44	NR	NR
Assal 1974 [23]^AP^	ReC	NR	230	NR	half of calculated dose given	within initial 4 hr
Keller 1975 [33]	CS	NR	345	NR	based on calculated dose	within initial 24 hr
Reddy 1977 [34]^P^	CS	≈ 0.6	NR	2.50	slow infusion till pH > 7.2	over mean of 4.9 hr
Lutterman 1979 [17]	ReC	1.4	167	NR	standard dose for all	within initial 6 hr
Lever 1983 [18]	ReC	NR	130-135^╫^	NR	NR	majority slow infusion
Hale 1984 [13]	RCT	1.3	150	NR	standard dose for all	over 1 hr
Morris 1986 [14]	RCT	NR	120	NR	titrated to pH, repeated till	intermittent dose, over
					pH > 7.15	30 min; 2 hr interval
Gamba 1991 [15]	RCT (DB)	≈ 7.5	84	NR	titrated to pH, repeated till	intermittent dose, over
					pH rise > 0.05	30 min; 2 hr interval
Okuda 1996 [19]	PrC	NR	200	NR	standard dose (50 mmol/hr)	over 4 hr
Green 1998 [24]^P^	ReC	NR	NR	2.08	NR	NR
Viallon 1999 [20]	ReC	1.4	120	NR	as per attending physician	over 1 hr
Latif 2002 [21]	ReC	NR	50	NR	standard dose for all	NR

CS = case series; PrC = prospective case-control; ReC = retrospective case-control; RCT = randomized controlled trial; DB = double-blind; Conc = concentration; NR = not reported; mM = mmol; Wt-adj = weight-adjusted

^†^Mean values provided separately for two study arms; ^╫^mean values provided separately for two study centers

^P^Pediatric studies; ^AP^mainly adults but including pediatric patients

Slow infusions using half-isotonic or isotonic preparations (approximately 1%) or small intermittent boluses of more concentrated preparations (approximately 8.4%) were preferentially used in later studies [13-15,17,18,20] to avoid too rapid pH or osmolality changes, with no evidence of risk or benefit with either methods.

### Primary outcomes

#### Duration of hospitalization

One single-center retrospective pediatric study assessed duration of hospitalization as an outcome measure [24]. Duration of hospitalization was significantly longer (87 vs. 67 hours, *p *= 0.01) for the bicarbonate group vs. children treated without bicarbonate. However, there was no adjustment for confounding variables. With multivariate analysis, duration of hospitalization was 23% longer in the bicarbonate group but did not reach statistical significance (*p *= 0.07). Using 29 pairs of matched patients (for calendar year, pH, and creatinine), duration of hospitalization was 37% longer in the bicarbonate group (*p *= 0.011).

In another brief report of 41 patients admitted for severe DKA, 5 patients had pH < 7.0 (mean 6.85 ± 0.09); only 4 received a small 50-mmol bolus of sodium bicarbonate, whereas 36 patients with pH > 7.0 (mean 7.15 ± 0.11) did not [21]. Bicarbonate therapy did not seem to have an impact on duration of hospitalization. Therefore, there may be a weak association with prolonged hospitalization in children with DKA treated with additional bicarbonate therapy, but the evidence is of very poor quality.

#### Mortality outcome

No published trials on the use of bicarbonate therapy in DKA were able to comment on any mortality difference with or without its use. Critically ill DKA cases with severe metabolic acidemia were excluded from most studies.

### Secondary outcomes (physiological)

#### Resolution of acidosis

Eight case-control studies have examined the rates of acidosis reversal with or without additional bicarbonate therapy, including three RCTs. The results are summarized in Table 3. Improvements in pH and serum bicarbonate levels were used as markers of acidosis reversal [13-15,17-20,24].

**Table 3:** Key studies on resolution of acidosis and ketosis with bicarbonate therapy in DKA

References	Trial design	No. of patients (bicarb vs. control)	Mean age (yr) and initial pH	Bicarbonate infusion	Control	Acidosis and ketosis
Hale et al. [13]	RCT	16 vs. 16	47 vs. 41	(1^st ^hr: 1 L isotonic saline for all)		Higher pH and bicarb levels at 2 hr
*Br Med J 1984*	(single center)		6.85 vs. 6.85	2^nd ^hr: 1 L isotonic bicarb vs.	1 L isotonic saline	in bicarb arm vs. control, *p *< 0.01
						BUT
				(3^rd ^hr: 1 L isotonic saline for all)		Slower decline in blood ketone in 1st hr in bicarb arm
						
Morris et al. [14]	RCT	10 vs.11	34 vs. 28	133.8 mmol if pH 6.9-6.99	no alkali	No difference in rate of change of pH, bicarb, ketones
*Ann Intern Med *1986	(single center)			OR 89.2 mmol if pH 7.0-7.09		OR time to reach pH 7.3
			7.03 vs. 7.00	OR 44.6 mmol if pH 7.1-7.14		OR bicarb levels to reach 15 mmol/L
				(over 30 min, 2 hourly until pH ≥ 7.15)		
						
Gamba et al. [15]	RCT	9 vs. 11	29 vs. 28	133.5 mmol/150 ml (pH 6.9-6.99)	0.9% saline, also	Higher pH at 2 hr in bicarb arm, *p *< 0.02
*Rev Inves Clin *1991	double-blind			89 mmol/100 ml (pH 7.0-7.09)	in similar aliquots	AND higher bicarb in bicarb arm, *p *< 0.01
	(single center)		7.05 vs. 7.04	44.8 mmol/50 ml (pH 7.1-7.14)		
				(over 30 min, repeated at 2 hr		Change in pH and bicarb larger in bicarb arm at 2 hr,
				if pH increase by < 0.05)		*p *< 0.01
						
Okuda et al. [19]	Prospective	3 vs. 4	24 vs. 34	50 mmol/hr over 4 hr	No alkali	Paradoxical increase in plasma acetoacetate in 1^st ^3 hr
*J Clin Endocrinol Metab 1996*	nonrandomized					in bicarb arm vs. control
	nonblinded		6.98 vs. 7.27	(IV insulin 0.1 U/kg/hr + 0.9% saline)		Increase in plasma 3-hydroxybutyrate level after bicarb
	(single center)		(*p *< 0.05)			ceased vs. steady decline throughout in control
Lutterman et al. [17]	Retrospective	12 vs. 12	41 vs. 34	167 mmol/L in 1 L	Low-dose insulin	No difference in mean pH rise in 1^st ^2 hr
*Diabetologia *1979	(single center)			over 1 hr (if pH ≤ 7.0)	IV 8 U/hr	OR mean time to reach pH ≥ 7.30
			6.89	(with high dose insulin		OR rate of decline of ketosis
				mean 260 U in 1st 6 hrs)		
						
Lever et al. [18]	Retrospective	52 (73 cases)	22.5-37.4 vs.	mean 130-135 mmol	No alkali	No difference in mean change in bicarb level per hr
*Am J Med *1983	(2 centers)	vs	24.5-48.0	(majority slow infusion)		OR mean change in pH per hr
		21 (22 cases)	6.94-7.00 vs.			
			6.89-7.07			
						
Viallon et al. [20]	Retrospective	24 vs. 15	45 vs. 47	mean 120 mmol (88-166)	No alkali	No difference in variation of mean pH, bicarb level, AG
*Crit Care Med *1999	(single center)			1.4% over 1 hr infusion		anion gap in 1st 24 hr
			6.93 vs. 7.00			OR mean time to reach pH > 7.30
						OR urine ketone clearance
						
Green et al[24]	Retrospective	57 (90 cases)	9.6 vs. 10.1	mean 2.08 mmol/kg (0.53-	No alkali	Unadjusted rate of bicarb rise faster in bicarb arm at
*Ann Emerg Med *1998	(single center)	vs		7.37 mmol/kg)		24 hr, *p *= 0.033
(pediatric)		49 (57 cases)	7.02 vs. 7.06			No difference in bicarb rise at 12 and 24 hr, or time to reach
						bicarb of 20 mmol/L (matched pair and multivariate analysis)

cases: DKA episodes; IV: intravenous; hr: hour; min: minutes; bicarb: bicarbonate

Two adult RCTs demonstrated biochemical benefit in terms of acidosis reversal time, with improved pH and bicarbonate levels at 2 hours of therapy in the bicarbonate arm. Of these, one study administered isotonic bicarbonate as a slow infusion [13], whereas the other administered small intermittent bicarbonate boluses of higher concentration titrated to severity of pH [15]. The latter study extended the follow-up duration to 24 hours of therapy and did not find a sustained biochemical benefit beyond 2 hours. A third adult RCT administered similar incremental small boluses of sodium bicarbonate but did not establish a similar biochemical advantage [14]. In addition, three retrospective adult studies [17,18,20] and one retrospective pediatric study [24] showed no improvement in acidosis resolution with use of bicarbonate therapy.

#### Resolution of ketosis

As shown in Table 3 two adult studies showed paradoxical worsening of ketonemia, including a slower decline in ketonemia in the first hour of bicarbonate infusion in a RCT [13], and an increase in plasma acetoacetate levels during the initial three hours of bicarbonate infusion in a small, prospective, nonrandomized study [19].

#### Insulin sensitivity and glycemic control

Results of pediatric and adult studies that reported insulin sensitivity and glycemic control as outcome measures are summarized in Table 4. No significant difference in rate of glucose decline or insulin requirement was demonstrated with bicarbonate treatment.

**Table 4:** Studies on insulin sensitivity and glycemic control

Reference	Trial design and size	Bicarb dose (intervention)	Insulin dose	Glycemic control
Hale et al. [13]	RCT	150 mmol	IM 20 U in 1st hr,	No difference in glucose decline over 2 hr
*Br Med J *1984	Adults (N = 32)	(standard)	6 U in both 2nd and 3rd hr	
				
Morris et al. [14]	RCT	120.4 mmol	Insulin 0.3 U/kg (IV + IM),	No difference in time for glucose to reach 250 mg/dL
*Ann Intern M *1986	Adults (N = 21)	(mean)	then IM 7 U/hr	No difference in total insulin required
				(1 hypoglycemia in control group)
				
Gamba et al. [15]	RCT	84 mmol	IV insulin 5 U/hr	No difference in glucose levels throughout 24 hrs
*Rev Cl In *1991	Adults (N = 20)	(mean)		No difference in total insulin required to reduce glucose
				to < 250 mg/dL, or till urine ketones were < 2+
				
Lutterman et al. [17]	Retrospective	167 mmol	High-dose insulin (mean	No difference in glucose decline in 1st 2 hrs
*Diabetologia *1979	Adults (N = 24)	(standard)	260 ± 60 U in 1st 6 hr)	No difference in mean glucose in 1st 8 hours
			vs. low dose 8 U/hr	(4 hypoglycemia in bicarb arm)
				
Lever et al. [18]	Retrospective	130-135 mmol	IM or IV insulin	No difference in glucose decline in 7 - 9 hrs
*Am J Med *1983	Adult (N = 73)	(standard)	5-6 U/hr (for all)	(2 hypoglycemia in bicarb arm)
				
Viallon et al. [20]	Retrospective	120 ± 40 mmol	IV insulin for all	No difference in normalization time of glycaemia
*Crit Care Med*1999	Adult (N = 39)	(mean)	(dose unspecified)	OR in mean quantity of insulin infused
				
Green et al. [24]	Retrospective	2.08 mmol/kg	IV insulin for all	No difference in insulin requirement in 24 hrs
*Ann Em Med *1998	Pediatrics (N = 106)	(mean)	(dose unspecified)	
				
Okuda et al. [19]	Prospective	200 mmol	IV 0.1 U/kg bolus insulin	No difference in glucose decline over 7 - 8 hrs
*J Clin En M *1996	Adults (N = 7)	(standard)	and then IV 0.1 U/kg/hr	

IM = intramuscular; IV = intravenous; U = units; bicarb = bicarbonate; L = liter; hr = hour

#### Potassium balance

Seven studies examined potassium balance as an outcome measure and are summarized in Table 5. One double-blind adult RCT, with mean bicarbonate dose of 84 ± 34 mmol, demonstrated lower serum potassium at 24 hours of therapy in the bicarbonate arm [15]. Another adult retrospective study, with mean bicarbonate dose of 120 ± 40 mmol, showed higher potassium supplementation in bicarbonate arm over 24 hours [20]. Four other studies (including one pediatric study) did not detect any statistical difference in the potassium balance [14,17,18,24].

**Table 5:** Studies on potassium balance and supplementation

Reference	Trial design and size	Bicarb dose (intervention)	Insulin dose	Potassium balance and supplementation
Morris et al. [14]	RCT	120.4 mmol	Insulin 0.3 U/kg (IV + IM),	No difference in serum K decline
*Ann Intern Med *1986	Adults (N = 21)	(mean)	then IM 7 U/hr	
				
Gamba et al. [15]	RCT	84 mmol	IV insulin 5 U/hr	Lower serum K at 24 hr for bicarb arm vs. control,
*Rev Cl In *1991	Adults (N = 20)	(mean)		*p *< 0.05
				BUT trend for more K given in control
				
Soler et al. [22]	Prospective	Grp 1: none	Grp 1: 234 U/24 hr	More K requirement over 24 hr for Grp 3
*Lancet *1972	Mixed (N = 25)	Grp 2: 200 mmol	Grp 2: 287 U/24 hr	Estimated 30 mmol/L of K needed for Grps 1 & 2,
(3-arm study; age 13-84 yr)		Grp 3: 400 mmol	Grp 3: 288 U/24 hr	& 40 mmol/L for Grp 3
*only 2 groups randomized*				(per L of fluid infused)
				
Lutterman et al. [17]	Retrospective	167 mmol	High-dose insulin (mean	No difference in mean serum K
*Diabetologia *1979	Adults (N = 24)	(standard)	260 ± 60 U in 1st 6 hr)	No difference in K requirement over 12 hrs
			vs. low dose 8 U/hr	
				
Lever et al. [18]	Retrospective	130-135 mmol	IM or IV insulin	No difference in K requirement
*Am J Med *1983	Adults (N = 73)	(standard)	5-6 U/hr (for all)	6 hypokalemia (< 3.3 mmol/L) in bicarb arm, 1 in control
				
Viallon et al. [20]	Retrospective	120 ± 40 mmol	IV insulin for all	More K requirement over 24 hr for bicarb arm,
*Crit Care Med*1999	Adults (N = 39)	(mean)	(dose unspecified)	*p *< 0.001
				1 hypokalemia (< 3 mmol/L) in bicarb arm
				
Green et al. [24]	Retrospective	2.08 mmol/kg	IV insulin for all	No difference in hypokalemia occurrence
*Ann Emerg Med *1998	Pediatrics (N = 106)	(mean)	(dose unspecified)	

Grp = group; IM = intramuscular; IV = intravenous; U = units; K = potassium; bicarb = bicarbonate; L = liter

A mixed adult and pediatric, three-arm prospective study, examined the association between mean cumulative bicarbonate doses and potassium requirement. The two groups that received saline and low-dose bicarbonate (mean 200 mmol) had comparable potassium supplementation during first 24 hours, whereas the third group with high bicarbonate dose (mean 400 mmol) received higher potassium supplementation [22].

#### Tissue oxygenation

One adult RCT reported a significantly slower rate of decline in blood lactate and lactate to pyruvate ratio in the bicarbonate treatment arm, compared with saline control, in the first hour of treatment in DKA [13]. A slow decline in blood lactate to pyruvate ratio was used to imply tissue hypoxia. A subsequent pediatric nonrandomized prospective study demonstrated that the initial decline of in vivo P_50 _(partial pressure of oxygen required to saturate 50% of the hemoglobin oxygen binding sites in a sample of whole blood) with DKA treatment was similar in both bicarbonate-treated group and controls. Bicarbonate therapy was not shown to affect oxygen transport adversely [16].

#### Cerebrospinal fluid acidosis

One adult RCT performed CSF analysis in approximately half of the adult patient cohort to investigate the concern of paradoxical CSF acidosis with bicarbonate administration. The study did not find any statistically significant difference in CSF pH and bicarbonate levels within 24 hours in the bicarbonate-treatment group and control. However, patient numbers were small, and a trend for larger decline in CSF pH at 6 to 8 hours was observed in the bicarbonate group [14]. In another nonrandomized study, the study subjects who received additional bicarbonate therapy for DKA [23] were compared with controls from an older study, which used the usual treatment with insulin and saline [39]. Both therapies induced a paradoxical drop in CSF pH after treatment for DKA, which was accompanied by a significantly higher CSF P_CO2 _and lesser increment in CSF bicarbonate level compared to blood, with no significant difference.

### Secondary outcomes (clinical)

#### Neurological deterioration and cerebral edema

The possible association of bicarbonate therapy with the development of CE in DKA was highlighted in three nonrandomized studies that investigated risk factors for CE in pediatric DKA patients (Table 6). Glaser et al. performed a multicenter, case-control study and identified 61 children with CE. Bicarbonate therapy was the only treatment variable associated with a greater risk of CE, after comparing with matched controls. The relative risk was 4.2 (95% confidence interval 1.5-12.1). Comparable proportions of children in the CE group and matched control had bicarbonate infused within 2 hours before neurological deterioration; hence no bias was detected [25]. Two other smaller studies found a trend for bicarbonate use and an association with CE, but the risk was not significant after adjusting for covariates, including baseline acidosis [26,27]. A fourth pediatric study demonstrated that impaired conscious level in DKA was associated with younger age and lower initial pH, and CE cases had lower pH compared with matched controls with no CE, at every conscious level studied [28]. No studies have examined CE risks in adult DKA population, in which CE has only been rarely reported [40-42].

**Table 6:** Studies on risk of cerebral edema in pediatric DKA population

References	Trial design	Case (children with CE)	Control(s)	Associated risks of CE	Bicarb therapy and CE risk
Glaser et al. [25]	Retrospective	N = 61	N = 174 (*matched*)	Higher urea nitrogen and lower arterial P_CO2 _levels	Bicarb therapy significantly a/w CE (matched control)
*NEJM *2001	case-control	Mean age: 8.9 yr	Mean age: 9.0 yr	at presentation (matched and random controls)	(23 of 61 with CE received bicarb;
	(multicenter)	Mean pH: 7.06	Mean pH: 7.09	and	vs. 43 of 174 matched controls);
	USA + Australia	(*matched for age, DM onset, pH/bicarb, glucose*)		smaller increase in Na+ (matched control)	RR 4.2 (*p *= 0.008)
			N = 181 (*random*)	and	
			Mean age: 11.3 yr	Younger age, newly dx DM, lower pH, higher	
			Mean pH: 7.12	glucose & Cr at presentation (random control)	
					
Lawrence et al. [26]	Prospective +	N = 21	N = 42 (*mostly random*)	Lower bicarb, higher urea, higher glucose levels	Trend towards association for bicarb therapy with CE
*J Pediatrics *2005	Retrospective	Mean age: 9.0 yr	Mean age: 9.6 yr	at presentation	(data for bicarb therapy available in 17 CE cases,
	case-control	Mean pH: 7.10	Mean pH: 7.20		with 34 random controls)
	(multicenter)	(*13 prospective*,	(*matched for institution*		
	Canada	*8 retrospective*)	*and data collection duration*)		
					
Edge et al. [27]	Prospective	N = 43	N = 169	Lower pH and/or lower bicarb levels, higher urea	Unadjusted OR of bicarb Rx for CE risk of 3.7 (*p *< 0.05)
*Diabetologia *2006	case-control	Mean age: 8.5 yr	Mean age: 8.9 yr	and potassium levels at presentation;	After adjustments for matching variables and baseline
	(multicenter)	Mean pH: 7.00	Mean pH: 7.20	more cumulative fluid volume given in 1st 4 hr,	acidosis, OR reduced to 1.5 (not significant)
	United Kingdom	(*matched for age, sex, DM onset, admission month*)		insulin administration in 1st hr, and higher quantity	
				of insulin given over 1st 2 hr	

DM = diabetes mellitus; bicarb = bicarbonate; Na+ = sodium; Cr = creatinine; CE = cerebral edema; neuro = neurological; RR = relative risk; OR = odds ratio; Rx = treatment

### Other neurological outcomes

Three adult studies have examined neurological recovery as a secondary outcome. One RCT examined mental status at 0, 2, 6, 12, and 24 hours after therapy, and found no difference in both treatment arms [15]. Two other retrospective studies also found no difference in neurological status with bicarbonate therapy, in patients with varying degrees of impaired mental status at baseline [18,20]. There were no pediatric studies on neurological recovery.

### Hemodynamic outcome

Three studies, including one RCT involving adult DKA patients with admission pH > 6.90, reported changes in clinical parameters, such as heart rate, respiratory rate, and mean arterial pressure as outcome measures. None reported any difference in clinical parameters with or without added use of bicarbonate [15,18,20].

## Discussion

### Summary of evidence

We conducted a systematic review of the literature, comparing additional use of bicarbonate infusion versus the usual treatment with insulin and hydration, in pediatric and adult patients with DKA. We have found marked heterogeneity and no clear evidence, with regards to the threshold for, concentration, amount, and timing of bicarbonate administration. In addition to such variability of treatment, there was retrospective evidence of clinical harm, such as increased risk for CE and prolonged hospitalization in children, and weak evidence of physiological harm, such as transient paradoxical worsening of ketosis and increased need for potassium supplementation. Theoretical benefits perceived with rapid acidemia reversal were not evident, apart from weak evidence of transient improvement in acidosis, with no evidence of any clinical efficacy.

### Physiological impact of bicarbonate therapy in DKA

The primary cause of acidemia in patients with DKA is ketoacidosis, with contribution from lactic acidosis and renal dysfunction. After metabolism of ketones during the recovery phase, bicarbonate is regenerated and aids the resolution of acidosis but is potentially affected by the development of hyperchloremia, which has been reported in more than 50% of adult and pediatric patients after 4 hours of therapy in DKA, and in more than 90% of patients by 8 to 20 hours [7,43]. It was observed and suggested in these studies that hyperchloremic acidosis is likely contributed by preferential renal excretion of ketones over chloride anion and volume repletion with saline, with the most rapid rise in hyperchloremia coinciding with the period of greatest saline administration [43]. Theoretically, adjunct use of bicarbonate administration may be more beneficial in the scenario of reduced renal bicarbonate genesis with concomitant acute kidney injury or in hyperchloremic acidosis where there is deficiency of bicarbonate relative to chloride.

Although bicarbonate therapy in DKA has been shown in two RCTs to improve acidosis resolution in the initial few hours of therapy, the comparator consisted of sodium chloride infusion. Thus, the initial favorable physiologic outcome with bicarbonate therapy might represent a reduced risk of hyperchloremic acidosis. Despite so, patient numbers were small, and this transient physiological benefit had not been demonstrated to persist beyond the initial 2 hours. Concerns were raised that bicarbonate therapy might interfere with tissue oxidation and with the clearance or renal excretion of ketones, hence accounting for the paradoxical worsening of ketosis.

Severe acidosis may inhibit the action of insulin on glucose utilization. Insulin resistance in humans has been shown to be higher at lower pH range and resistance to fall steeply at pH above 7.2 [44]. Early and rapid correction of acidemia can theoretically increase insulin sensitivity. However, as discussed, there is no evidence of the above-postulated benefit of bicarbonate therapy. Instead, lower serum potassium and increased need for potassium supplementation had been demonstrated by mainly adult studies, including one small RCT, in the bicarbonate treatment arm. Although no fatal outcomes or arrhythmias had been reported as a result of hypokalemia, it would be prudent to pay close attention to this anticipated complication.

Acute reversal of acidemia with bicarbonate also has been linked to worsening of tissue hypoxia. Acidosis induces a mild increase in P_50 _and reduced hemoglobin-oxygen affinity (Bohr effect), but at the same time is associated with lower levels of 2,3-diphosphoglycerate (2,3-DPG) in erythrocytes [45], which leads to a counteractive increased hemoglobin-oxygen affinity. In the initial presentation of DKA, a fine balance exists in favor of the former (Bohr effect) [16], which can theoretically be disrupted by rapid treatment of acidemia, as 2,3-DPG levels were demonstrated to remain strikingly low for days despite improvement in acidosis [46], resulting in net increase in hemoglobin-oxygen affinity and impaired tissue oxygenation. However, this phenomenon is generally seen in the initial treatment phase of DKA, regardless of bicarbonate therapy. P_50_, along with blood lactate to pyruvate ratio, are merely surrogate markers of peripheral tissue oxygenation used in studies. Therefore, there remains to be insufficient evidence that additional bicarbonate administration affects tissue oxygenation adversely.

Bicarbonate therapy in patients with DKA appeared to be associated with increased obtundation and profound cerebrospinal fluid (CSF) acidosis in an early study [47]. A possible explanation for this observation may be the preferential movement across the blood-brain barrier of CO_2 _compared with bicarbonate during treatment of DKA, when both P_CO2 _and bicarbonate levels rise in the blood. It was postulated that rapid reversal of acidemia with bicarbonate might promote paradoxical CSF acidosis and contribute to adverse neurological outcomes. However, we have not found any evidence that bicarbonate infusion causes increased paradoxical CSF acidosis compared with conventional DKA treatment.

In essence, most of the theoretical biochemical gains and harm with bicarbonate administration were not evident in actual case scenarios, and the overall physiological impact with such treatment is dismal.

### Clinical impact of bicarbonate therapy in DKA

CE followed by coma is a devastating complication of DKA, with an incidence of 1% and mortality of 24% [25,27], and appears to be essentially exclusive to children and young adolescents [48]. The pathophysiology of CE remains unclear, and a detailed discussion on this is beyond the scope of this article. In essence, possible mechanisms include initial cerebral vasoconstriction and reduced cerebral blood flow from acidosis and hypocapnia, cytotoxic edema, and cerebral injury, followed by cerebral hyperemia, reperfusion injury, and vasogenic edema, coupled with increased blood brain barrier permeability, during the rehydration phase of DKA [48,49]. Several reports of sudden death following irreversible coma in children and young adults with DKA were published in the 1960s, including development of diabetes insipidus in some, with postmortem findings of CE and neuronal degeneration [50-52].

From our earlier discussion, it is apparent that cerebral function in DKA is related to severity of acidosis, even when there is no occurrence of CE. There were no details on the reasons for bicarbonate administration in previously mentioned studies on CE in children with DKA, and it would be logical to assume that those who were given bicarbonate were likely to have more severe DKA or even circulatory collapse, factors which by themselves might predispose to adverse neurological outcomes. It should be noted that studies on risk factors for CE were based on historical cases, when the use of bicarbonate frequently accompanied high-dose insulin protocols, where the combination of both might have theoretically worsened the risk of CE.

Apart from the risk of CE, we also have discussed the retrospective evidence that bicarbonate therapy is associated with prolonged hospitalization in the pediatric DKA cohort. Such studies were again subjected to the natural confounder that children admitted with a lower arterial pH (who were potentially more ill) and in earlier study years were more likely to be given sodium bicarbonate. On the other hand, there is no evidence that the rapid reversal of acidemia with bicarbonate therapy improves any clinical outcome, especially in the pediatric cohort. Documentation of improved mental status from initial diabetic coma following treatment (including bicarbonate therapy) came only from pediatric and adult case reports and series [23,29,30,36]. It could not be ascertained, however, if a favorable neurological outcome was attributable to the use of initial bicarbonate therapy.

In addition, there is no evidence of improved hemodynamic stability with the use of bicarbonate administration in DKA. Much of the perceived benefit in acute reversal of severe acidemia is only based on animal and experimental studies, which demonstrated weakened end-organ response to catecholamines at pH < 7.2, with bradycardia, negative inotropism, impaired cardiac output, peripheral vasodilatation, and refractory hypotension [53]. Therefore, even though the clinical harm with bicarbonate treatment is merely an association (and not causation), the lack of clinical benefits does not justify its routine use especially in children.

### Limitations of studies

In general, patient numbers in the three adult RCTs were small and lacked the statistical power to examine clinical outcomes. Most prospective trials excluded patients with severe concomitant illnesses, in whom the adverse cardiovascular effects of severe acidemia are believed to be more significantly seen. There were no trials performed in the scenario of more severe acidemia (pH < 6.85), and it seems unlikely that such studies will be performed. Understandably, documentation of presumed benefits of bicarbonate rescue in cases of DKA presenting with more severe acidemia and cardiovascular collapse or significant hemodynamic compromise were confined to case reports [32,35,36]. Clinical judgment, opinion, and expertise prevail in such circumstances in the absence of trials. There are a paucity of data on bicarbonate administration in the pediatric DKA population with no randomized trials performed, forcing the extrapolation of adult data, despite the likelihood that the pathophysiology in both cohorts are fundamentally different. Studies that report clinical harm with bicarbonate treatment in children are all retrospective in design and subjected to the various confounders as discussed earlier.

There were limited DKA trials during the past decade, especially in the context of modern day emergency medicine or intensive care. The data of the past decade were mostly focused on the adverse neurological outcome of bicarbonate treatment in the pediatric DKA population. There is increasing recognition of the development of hyperchloremic acidosis for the treatment phase of DKA with fluid resuscitation, which might impact the resolution of acidemia [54]. The clinical effects of hyperchloremic acidosis remain uncertain.

## Conclusions

The evidence to date does not support the use of bicarbonate administration for the emergent treatment of DKA, especially in the pediatric population, in view of possible clinical and physiological harm and the lack of clinical or sustained physiological benefits. There also is insufficient evidence to justify the recommendation of bicarbonate administration in more extreme acidemia of pH < 6.90. Future research should focus on the use of more balanced and physiological resuscitation fluids with buffering capacity, in the modern context of DKA management, with the goal of reducing the component of hyperchloremic acidosis in DKA while minimizing the risk of CSF acidosis and associated CE.

## Competing interests

The authors declare that they have no competing interests.

## Authors' contributions

RB and HRC conceived the topic review idea and proposal. HRC and AS performed the literature search and selected the relevant articles for inclusion independently. RB adjudicated any disagreements in article inclusion. HRC, AS, and RB reviewed the selected articles in entirety. HRC and RB wrote the initial draft of the manuscript. All authors reviewed and edited the manuscript.
